# The Impact of Regular Exercise on Life Satisfaction, Self-Esteem, and Self-Efficacy in Older Adults

**DOI:** 10.3390/bs13090714

**Published:** 2023-08-28

**Authors:** Turhan Toros, Emre Bulent Ogras, Ali Burak Toy, Abdulaziz Kulak, Huseyin Tolga Esen, Sevket Cihat Ozer, Talip Celik

**Affiliations:** 1 Department of Coaching Education, Mersin University, Mersin 33000, Turkey; 2 Faculty of Sport Sciences, Mersin University, Mersin 33000, Turkey; 3 Physical Education and Sports School, Ardahan University, Ardahan 75000, Turkey; 4 Physical Education and Sports School, Harran University, Sanliurfa 63300, Turkey; 5 Faculty of Sport Sciences, Akdeniz University, Antalya 07070, Turkey; 6 Faculty of Sport Sciences, Nevsehir Hacı Bektas Veli University, Nevşehir 50000, Turkey; 7 Talip Celik, Malatya Vocational School, Inonu University, Malatya 44000, Turkey

**Keywords:** physical exercise, older adults, life satisfaction, self-esteem, self-efficacy, aging, mental health, lifestyle

## Abstract

This study employed the correlational survey model to examine how regular exercise influenced life satisfaction, self-esteem, and self-efficacy in men over 65. The study sample included a total of 215 participants, of whom 110 exercised regularly (for at least 45 min, three times a week), while 105 engaged in no physical exercise. Regular exercisers were found to score significantly higher on life satisfaction, self-esteem, and self-efficacy scales as compared to non-exercisers. These scores also increased significantly with age and prolonged exercise history. A moderate and positive correlation was detected between life satisfaction, self-esteem, and self-efficacy among regular exercisers, while non-exercisers showed low to moderate correlations. The findings suggest that regular exercise can enhance life satisfaction, self-esteem, and self-efficacy in the elderly male population. Such effects appear to be associated with greater age and lifetime exercise history, highlighting the value of regular physical exercise in improving the quality of life among older adults.

## 1. Introduction

Life satisfaction, self-esteem, and self-efficacy are important psychological factors for individuals over 65. Life satisfaction refers to how content one is with their overall life [1], self-esteem to how much one values themselves [2], and self-efficacy to how confident one is in their own abilities and capabilities [3]. These factors may shape an individual’s quality of life. Exercise, on the other hand, is vital for physical and mental health in this age group [4]. Life satisfaction, self-esteem, and self-efficacy are important psychological factors for individuals over 65. Life satisfaction refers to how content one is with their overall life [1], self-esteem to how much one values themselves [2], and self-efficacy to how confident one is in their own abilities and capabilities [3]. These factors may shape an individual’s quality of life. Exercise, on the other hand, is vital for physical and mental health in this age group [4]. Regular exercise, in a broad sense, refers to any planned, structured, repetitive, and purposeful physical exercise performed with the aim of improving or maintaining one or more aspects of physical fitness and health. While the specific type, intensity, duration, and frequency of the exercise can vary greatly depending on the individual’s age, physical condition, and specific health goals, regular exercise typically implies a consistent, long-term commitment to an active lifestyle. The relationship between regular exercise and life satisfaction, self-esteem, and self-efficacy in older individuals is complex and may depend on various variables [5].

Life satisfaction is a holistic measure of how content individuals are with their overall lives [1]. It includes satisfaction with specific life domains—such as health, family, work, and education—and general life satisfaction. Satisfaction with certain domains of life like work and family contributes to one’s overall satisfaction with life. Various factors influence this evaluation, such as past experiences, current situation, and future aspirations [6]. In particular, alignment between one’s current situation and future goals influences their satisfaction with life [7]. This implies that life satisfaction is a subjective judgment of how happy one is with their life. Life satisfaction is a common indicator of quality of life, as it allows people to assess their lives [8]. However, self-esteem, which is one’s self-evaluation of their abilities and values, is also important for understanding quality of life.

Self-esteem is a self-evaluation based on how individuals perceive their own worth and abilities [2]. It reflects whether individuals assess themselves positively or negatively and is generally related to their belief in and respect for themselves. Rosenberg proposed that self-esteem indicates how individuals view themselves, especially how they value themselves and how positive or negative their self-worth is [2]. Self-esteem may affect an individual’s social relationships, work performance, life satisfaction, and mental health. For example, high self-esteem is associated with greater life satisfaction, better job performance, and improved mental health [9]. On the other hand, low self-esteem is often linked to depression, anxiety, and lower life satisfaction [10]. Therefore, self-esteem can have a significant impact on an individual’s quality of life and overall health. In addition to self-esteem, self-efficacy, which is one’s belief in their own capabilities and skills, also matters for understanding quality of life.

Self-efficacy is an individual’s belief in their ability to accomplish a specific task. This concept has been extensively studied by Albert Bandura, who found that self-efficacy beliefs can significantly influence individuals’ behaviors, thoughts, motivations, and emotional reactions [3]. For instance, those with high self-efficacy beliefs are more likely to persist in the face of adversity and make greater efforts to overcome challenges. Self-efficacy can also have a significant impact on individuals’ overall satisfaction with life and quality of life, meaning that people with greater self-efficacy beliefs tend to experience higher levels of life satisfaction and a better quality of life [11]. Additionally, they are more likely to demonstrate greater resilience when faced with difficulties and may be more successful. Therefore, self-efficacy beliefs can have a significant impact on an individual’s quality of life and overall health.

In old age, psychological factors such as life satisfaction, self-esteem, and self-efficacy significantly impact overall health and quality of life. The elderly, or senior citizens, often encounter physical health changes and various challenges in different areas of life, which can have an impact on these factors. Exploring the interplay between these factors and their association with regular exercise holds promise for improving the well-being and health outcomes of this age group.

This study advances the literature by examining the correlation between regular physical exercise and key psychological variables—life satisfaction, self-esteem, and self-efficacy—within the under-represented demographic of men over 65. Despite acknowledged exercise benefits on these factors, our understanding within this age group remains limited, necessitating this investigation. The exploration of the long-term exercise’s potential cumulative benefits further enriches our understanding of the nexus between physical exercise and mental health. By filling this critical literature gap, the study bolsters the empirical foundation for public health initiatives that promote physical exercise among older adults. Therefore, this research not only enhances the scholarly discourse on exercise and mental health among the aging population but also has significant practical implications for health policy and intervention design, ultimately aiming to foster a healthier and more prosperous society.

To accomplish the research objective, the study addressed the following research questions:

1. Do older adult males (aged 65+) with regular exercise habits have a significantly different level of life satisfaction than those with no regular exercise habits?
2. Do older men who exercise regularly have significantly higher levels of self-esteem than those who do not?
3. Do self-esteem levels of older adults doing regular exercise differ significantly from those of the same age group doing no physical exercise?
4. Do the levels of life satisfaction, self-esteem, and self-efficacy vary significantly among older adults over 65 years old, depending on whether or not they engage in regular exercise?
5. Is there a significant difference in the levels of life satisfaction, self-esteem, and self-efficacy among older adults who exercise regularly, depending on their lifetime exercise history?
6. Is there a significant relationship between the levels of life satisfaction, self-esteem, and self-efficacy in older adults over 65 years old, regardless of whether or not they exercise regularly?

## 2. Material and Methods

This study aimed to investigate the correlation between life satisfaction, self-esteem, and self-efficacy in older adults, aged 65 or above, by comparing participants who engaged in regular exercise with those who did not. A relational screening model was used to analyze the data. This technique is typically used to identify trends and patterns in the data, rather than to establish causal relationships [12]. Simple random sampling was used to select the participants for the study in order to ensure that each participant has an equal chance of being selected and to reduce the risk of sampling bias, thus increasing the likelihood that the conclusions and inferences will apply to the target population. However, simple random sampling can be costly and time-consuming, especially for large populations. Additionally, in small and/or dispersed populations, accessing all population units may be difficult, making simple random sampling more complex [13].

### 2.1. Participants

The present study recruited a sample of 215 males aged above 65 years, with a mean age of 67.45 ± 7.76 years. Participants were selected using a simple random sampling method from the pool of individuals who volunteered. A significant metric employed in the characterization of this group was the Body Mass Index (BMI), as defined by the World Health Organization. The BMI is derived from the weight (in kilograms) of an individual divided by the square of their height (in meters) (BMI = kg/m^2^). The established classifications for adult BMI are:

Underweight: <18.5

Normal Weight: 18.5–24.9

Overweight: 25.0–29.9

Obesity: ≥30.0

All participants in this study had a BMI value within the range of 24 and above. Regarding their physical exercise, 110 males engaged in jogging for a minimum of three days per week, with each session lasting at least 45 min. The intensity of these sessions was modulated to account for approximately 60–70% of the participant’s maximal heart rate. These sessions were meticulously supervised by a team of three sports science specialists to ensure adherence to the specified intensity and duration. Any deviations observed during the sessions prompted immediate corrective feedback from the specialists. Conversely, the other 105 participants did not partake in any structured physical exercises.

### 2.2. Data Collection Tools

#### 2.2.1. Personal Information Form

The researchers developed a form to gather personal data from the participants. The form included questions about their regular exercise habits, age, and lifetime exercise history.

#### 2.2.2. Satisfaction with Life Scale (SWLS)

The SWLS is a five-item questionnaire developed by Diener et al. [1]. Participants rate their agreement with each item on a seven-point scale, ranging from ‘strongly disagree’ to ‘strongly agree’. The Turkish language version of the SWLS was adapted by Yetim [14], who reported a test–retest reliability of 0.58 over four- and six-year periods. The adaptation study also reported an internal consistency of 0.78 [14].

#### 2.2.3. Coopersmith Self-Esteem Inventory (CSEI)

The CSEI is a 25-item self-assessment scale developed by Coopersmith for use in the adult population [15]. Participants respond to each item on a two-step response scale, with higher scores indicating higher self-esteem. The lowest possible score is 0, and the highest possible score is 100. The validation and reliability study of the CSEI for the Turkish language was conducted by Turan and Tufan [16].

#### 2.2.4. Self-Efficacy Scale (SES)

The SES is a 10-item scale designed by Riggs et al. [17] to measure individuals’ confidence in their abilities. The original scale was adapted for Turkish culture by Öcel [18], and the Turkish language version also contains 10 items and a single structure. The scale is scored on a 5-point Likert scale, with higher scores indicating stronger self-efficacy beliefs. The highest possible score on the SES is 50, and the lowest is 10. The internal consistency of the scale was found to be 0.86 in the original study [18]. However, the factor analysis performed during the scale’s adaptation study revealed that the scale has a single-factor structure with item factor loading values ranging between 0.32 and 0.85. The internal consistency of the scale in the adaptation study was 0.61 [18]. 

### 2.3. Data Analysis

A statistical software package was utilized to analyze the research data. The Cronbach’s alpha reliability coefficient assessed the internal consistency of the research scales. The kurtosis and skewness values checked the suitability of parametric tests for this analysis. The *t*-test compared two groups, and One-Way ANOVA compared more than two groups. Pearson’s correlation analysis followed the relational model, and the significance level was 0.05.

## 3. Results

Below are the research findings along with their corresponding explanations, organized according to the sub-problems that were examined.

The results of the *t*-test in Table 1 show that there was a significant difference in the mean life satisfaction scores of individuals who exercised regularly and those who did not exercise. Men who exercised regularly at age 65 or above had higher life satisfaction scores than men who did not exercise (t = 3.911, *p* < 0.05).

A statistically significant difference was found between the mean self-esteem scores of men aged 65 and above who exercised regularly and those who did not exercise, as shown in Table 2. The mean self-esteem scores of the exercisers were significantly higher than those of their counterparts who did not exercise (t = 4.746, *p* < 0.05).

The *t*-test results in Table 3 show that there is a statistically significant difference in the mean self-efficacy scores of individuals aged 65 years and above who exercised regularly and those who did not exercise. This means that there is a high probability that the difference in mean self-efficacy scores between the two groups is not due to chance. The mean self-efficacy score of regular exercisers was significantly higher than that of non-exercisers (t: 3.952; *p* < 0.05).

Table 4 presents data regarding the mean life satisfaction, self-esteem, and self-efficacy scores of regular exercisers, divided into three age groups. The analysis results revealed statistically significant differences in the mean scores between these age groups. Specifically, individuals aged 69 and above had higher mean life satisfaction (SWLS) scores than those aged 65–66. Exercising men aged 67–68 had higher mean scores in self-esteem (CSEI) and self-efficacy (SES) than those aged 65–66. Additionally, participants aged 69+ had higher mean self-efficacy scores than those aged 65–66.

Table 5 compares the mean life satisfaction, self-esteem, and self-efficacy scores of men who did not engage in physical exercise, divided into three age groups. Data analyses found no significant differences in the mean scores between the age groups (*p* > 0.05), which means that the mean life satisfaction (SWLS), self-esteem (CSEI), and self-efficacy (SES) scores of men who did not engage in physical exercise were similar across the three age groups. 

Table 6 presents a comparison of the mean scores for life satisfaction, self-esteem, and self-efficacy in men who engaged in regular exercise, categorized by their lifetime exercise history in years. The results showed statistically significant differences in the mean scores between the groups classified according to exercise history. Specifically, individuals who had been exercising for 40 years or more had greater mean life satisfaction (SWLS) scores than those who had been engaging in regular physical exercise for 1 to 20 years. Participants exercising for 21 to 40 years had greater mean scores in self-esteem (CSEI) and self-efficacy (SES) than those who exercised for 1 to 20 years. Additionally, individuals with 40+ years of lifetime exercise history had higher mean self-efficacy scores than those exercising for 1 to 20 years.

Table 7 shows the results of a correlational analysis examining the association between life satisfaction, self-esteem, and self-efficacy in individuals aged 65 years and above who engage in regular exercise. The results showed that there were significant, positive, and moderate relationships between all three variables. Specifically, the correlation between life satisfaction and self-esteem was moderate and positive, such that individuals with higher life satisfaction also had higher self-esteem (r = 0.680, *p* < 0.05). The correlation between life satisfaction and self-efficacy was 0.698 (*p* < 0.05). This means that there was a moderate positive relationship between these two variables, meaning that exercisers with higher life satisfaction also had higher self-efficacy. Another significant, positive, and moderate relationship was detected between self-esteem and self-efficacy (r = 0.581, *p* < 0.05) among men aged 65 or above engaging in regular physical exercise.

Correlational analyses examining the association between life satisfaction, self-esteem, and self-efficacy scores of non-exercisers showed significant, positive, and moderate relationships between life satisfaction and self-esteem (r = 0.306, *p* < 0.05), and between life satisfaction and self-efficacy (r = 0.308, *p* < 0.05). However, the relationship between self-esteem and self-efficacy was only significant and positive, but of low-level (r = 0.194, *p* < 0.05) among men doing no exercise (Table 8).

## 4. Discussion 

This research aimed to examine the correlation between life satisfaction, self-esteem, and self-efficacy in older men, comparing those who reported doing exercise regularly with those reporting no physical exercise. The analysis of the research results supports a large body of existing academic work, showing significant differences in life satisfaction between elderly men who engage in regular physical exercise and those who do not. Many studies have highlighted the role of regular physical exercise in life satisfaction [19]. Physical exercise is known for its ability to improve physical well-being and help manage chronic health conditions [20]. Moreover, regular physical exercise has been linked to lower stress, better sleep quality, and higher mood and life satisfaction [21]. In the group of individuals over 65, the effect of physical exercise is mainly seen in its contribution to overall health and quality of life [22]. Aging involves various physical and mental changes, but these changes can be reduced by regular physical exercise [23]. Empirical evidence has shown the positive impact of regular physical exercise on life satisfaction scores, especially in individuals over 65 years old [24]. Therefore, the research results confirm the benefits of physical exercise on life satisfaction, emphasizing its importance, especially in older individuals. The higher life satisfaction scores in individuals who exercise regularly compared to those who do not validate the value of physical exercise in enhancing life satisfaction.

The finding of a significant difference in self-esteem scores between participants who engaged in regular exercise and those who did not seems to be consistent with the findings of previous research. Several studies have shown that regular physical exercise has a beneficial effect on self-esteem and self-concept [25]. This suggests that the benefits of exercise extend beyond physical health and can also impact how individuals perceive and evaluate themselves. More specifically, the positive correlation between regular physical exercise and self-esteem in the older adult population may be a marker of their ability to proactively manage and navigate their lives [26]. Individuals who lead physically active lifestyles generally have greater self-esteem, which in turn has a positive impact on their quality of life and overall satisfaction with life [27]. The results of this study corroborate the beneficial effects of regular physical exercise on self-esteem. The evidence that self-esteem scores were higher in individuals who consistently engaged in physical exercise as compared to those who did not highlights the vital role that physical exercise plays in enhancing self-confidence and self-worth in this population. In that regard, the findings of this study contribute to the existing literature on the relationship between regular physical exercise and self-esteem, and the results suggest that regular physical exercise may be a viable intervention for improving self-esteem in older adults.

The current study showed that self-efficacy scores were significantly higher for individuals who engaged in regular physical exercise than for those who did not exercise. This finding is consistent with the results of previous research, which have shown that physical exercise can positively affect an individual’s perceptions of their self-efficacy [28]. Additionally, there is evidence to suggest that regular exercise plays a particularly important role in enhancing self-efficacy in older adults [29]. This may be because exercise can help older adults develop a more positive outlook on their own abilities, as well as improve their ability to manage daily activities and cope with challenges [30]. The findings of this study provide valuable insights into the potential of exercise to improve self-efficacy in older adults, which should be taken into consideration when developing exercise programs and other strategies geared towards improving the quality of life among senior citizens.

The analyses also revealed that life satisfaction, self-esteem, and self-efficacy scores increased with age among our participants. This finding is in agreement with previous research, which has shown that aging can have a positive impact on these three areas of well-being [31,32,33]. One possible explanation for this is that older adults have more experience in self-acceptance and coping with life’s challenges, which could allow them to develop a more positive outlook on life and a stronger sense of self-worth [30]. Regular physical exercise has also been shown to have a positive effect on life satisfaction, self-esteem, and self-efficacy. This suggests that regular exercise may further enhance these areas of well-being in older adults [22,23,24,25,26,27,28,29,30,31,32,33,34]. Such insights can prove valuable in designing interventions that promote the quality of life for them.

No significant differences were detected in the mean scores of life satisfaction, self-esteem, and self-efficacy among non-exercisers, regardless of age group. This finding is consistent with the results of other recent studies, which have also found that physical exercise is a significant predictor of these psychological aspects. The absence of a significant increase in life satisfaction, self-esteem, and self-efficacy levels among non-exercising individuals may negatively impact their overall quality of life. [35]. This is because these psychological aspects are important for well-being and can contribute to a sense of purpose, meaning, and satisfaction in life [36,37]. The findings of this study underscore the importance of health policies and programs designed to promote regular physical exercise among older adults, and such interventions could help improve the psychological well-being of older adults and promote their overall quality of life. 

Greater lifetime exercise background was found to cause a significant improvement in life satisfaction, self-esteem, and self-efficacy scores among men over 65. These results are supported by several recent studies. For example, previous research showed that the length of exercise history had a positive effect on self-esteem and life satisfaction [22]. Also, some suggested that the theory of self-efficacy implies that individuals’ confidence in their ability to maintain physical exercise might help keep the habit of regular exercise [11]. This observation further strengthens the effect of exercise on life satisfaction, self-esteem, and self-efficacy.

The data analyses also revealed a significant and positive correlation between regular physical exercise and self-esteem, self-efficacy, and life satisfaction in men over 65. This finding is consistent with the results of previous work in the literature, which has also found that these three factors are interrelated. One such study found a relationship between self-esteem and both self-efficacy and life satisfaction. Moreover, a positive link was found between self-efficacy and life satisfaction [38]. These findings agree with our results, showing that these three factors interact similarly in older individuals who exercise regularly. Also, regular exercise is known to positively affect self-esteem and life satisfaction [39]. This study showed that older men doing regular exercise had greater levels of life satisfaction and self-esteem. Therefore, it can be said that the positive effect of exercise on these three factors contributes to the overall health and quality of life in this population. These findings highlight the connection between life satisfaction, self-esteem, and self-efficacy, which are important for older individuals’ tendency to keep regular physical activities [40,41,42]. Another significant but lower correlation was found between life satisfaction, self-esteem, and self-efficacy in non-exercising men over 65. All these findings suggest that life satisfaction, self-esteem, and self-efficacy are key factors when evaluating such attributes in both exercising and non-exercising older individuals.

Previous research has underscored the significance of exercise in enhancing the quality of life among adults aged 65 and above, yet the findings primarily focus on older adults who engage in regular physical exercise within this specific age group. More research is needed to explore the association between exercise patterns and quality of life across a wider range of adult age groups. Furthermore, the study revealed that scores for life satisfaction, self-esteem, and self-efficacy varied based on the length of exercise history or background among men who engaged in regular exercise. This observation provides valuable insights for future research aiming to examine the precise influence of exercise history on quality of life in more depth. Such research could contribute to identifying specific factors that contribute to the benefits of exercise, including the duration and intensity of physical exercise. 

## 5. Conclusions

Our study yielded salient findings regarding the psychological benefits of regular exercise in individuals aged 65 and above. Participants who engaged in routine physical activity demonstrated markedly enhanced scores in life satisfaction, self-esteem, and self-efficacy compared to their sedentary counterparts. These scores exhibited a notable ascendancy in tandem with participants’ age and the duration of their exercise engagement. Furthermore, a significant positive correlation was discerned between life satisfaction, self-esteem, and self-efficacy among the active participants. In contrast, the non-exercising cohort presented a more muted correlation between these psychological measures. Intriguingly, our age-grouped analysis did not identify any pronounced variance in these scores across different age brackets within the inactive group, suggesting the presence of alternative factors modulating their perceived quality of life.

This study, however, is not devoid of limitations. One notable gap was the lack of consideration given to participants’ medicinal intake, notably the usage of antidepressants, which can substantially modulate mood and well-being perceptions. This becomes especially germane when gauging psychological constructs, such as life satisfaction, self-esteem, and self-efficacy. It is imperative for subsequent studies to adopt an integrative lens, one that encompasses not only the experiential facets of individuals in this age category but also duly incorporates broader pharmacological influences. Such a holistic analysis would pave the way for a nuanced understanding of quality-of-life determinants for seniors.

Drawing upon the data derived from our study, it might be prudent to suggest the introduction of physical routines with reduced intensities for elderly participants, particularly those demonstrating limited physical stamina. It might be feasible to contemplate a flexible exercise paradigm, potentially comprising three intervals of 15 min, culminating in an aggregate duration of 45 min. Activities embodying a rhythmic quality and demanding extensive muscle group involvement, such as walking, jogging, dancing, swimming, and cycling, could be recommended as especially advantageous. The findings further hint at the potential importance of jogging, advocating for durations not less than 45 min, executed no less than three times per week, as potentially integral to amplifying the overall well-being and life quality in elderly groups.

In essence, the study unequivocally accentuates the intrinsic linkage between consistent physical activity and amplified scores in life satisfaction, self-esteem, and self-efficacy among the elderly. A profound grasp of the multifarious advantages and the historical precedence of regular exercise equips healthcare professionals and policymakers with the requisite knowledge to curate decisions fostering enriched well-being and promoting an optimized aging trajectory for senior populations.

## Figures and Tables

**Table 1:** Mean Satisfaction with Life Scale (SWLS) Scores of Participants by Regular Exercise Status.

Scale	Groups	N	Mean	SD	t	*p*
SWLS	Regular Exercisers	110	6.06	0.84	3.911	0.00 *
	Non-Exercisers	105	3.02	1.72		

* *p* < 0.01.

**Table 2:** Mean Coopersmith Self-Esteem Inventory (CSEI) Scores of Participants by Regular Exercise Status.

Scale	Groups	N	Mean	SD	t	*p*
CSEI	Regular Exercisers	110	74.25	0.98	4.746	0.00 *
	Non-Exercisers	105	46.86	1.06		

* *p* < 0.01.

**Table 3:** Mean Self-Efficacy Scale (SES) Scores of Participants by Regular Exercise Status.

Scale	Groups	N	Mean	SD	t	*p*
SES	Regular Exercisers	110	3.94	1.49	3.952	0.00 *
	Non-Exercisers	105	1.37	1.99		

* *p* < 0.01.

**Table 4:** Comparison of SWLS, CSEI, and SES Scores of Participants Doing Regular Exercise by Age Variable.

Scales	Age Groups		N	Mean	SD	F	*p*	Tukey
SWLS	65–66	A	41	4.61	0.94	4.390	0.00	A < C
	67–68	B	36	5.24	1.43			
	69+	C	33	5.99	1.35			
CSEI	65–66	A	41	60.75	1.24	4.960	0.00	A < B
	67–68	B	36	75.12	1.36			
	69+	C	33	63.04	0.006			
SES	65–66	A	41	3.03	0.732	8.700	0.00	A < B A < C
	67–68	B	36	4.61	0.900			
	69+	C	33	4.91	0.698			

**Table 5:** Comparison of SWLS, CSEI, and SES Scores of Non-Exercisers by Age Variable.

Scales	Age Groups		N	Mean	SD	F	*p*	Tukey
SWLS	65–66	A	38	4.96	1.63	0.723	0.48	--
	67–68	B	35	5.09	0.227			
	69+	C	32	4.35	0.220			
CSEI	65–66	A	38	52.03	0.179	1.400	0.26	--
	67–68	B	35	51.63	0.173			
	69+	C	32	51.65	0.208			
SES	65–66	A	38	3.00	0.125	0.336	0.71	--
	67–68	B	35	2.86	0.097			
	69+	C	32	3.00	0.140			

**Table 6:** Comparison of SWLS, CSEI, and SES Scores of Participants doing Regular Exercise by Lifetime Exercise History.

Scales	Exercise History		N	Mean	SD	F	*p*	Tukey
SWLS	1–20 years	A	44	4.06	1.65	4.350	0.00	A < C
	21–40 years	B	38	5.11	1.39			
	40+ years	C	28	5.62	1.49			
CSEI	1–20 years	A	41	60.71	1.20	4.940	0.00	A < B
	21–40 years	B	36	75.19	1.91			
	40+ years	C	33	63.48	0.048			
SES	1–20 years	A	41	3.38	0.620	8.720	0.00	A < B A < C
	21–40 years	B	36	4.09	0.671			
	40+ years	C	33	4.86	0.600			

**Table 7:** Correlation between Life Satisfaction, Self-Esteem, and Self-Efficacy Scores of Regular Exercisers.

Scales	SWLS	CSEI	SES
SWLS	1		
CSEI	0.680 **	1	
SES	0.698 **	0.581 **	1

** *p* < 0.05.

**Table 8:** Correlation between Life Satisfaction, Self-Esteem, and Self-Efficacy Scores of Non-Exercisers.

Scales	SWLS	CSEI	SES
SWLS	1		
CSEI	0.306 **	1	
SES	0.308 **	0.194 **	1

** *p* < 0.05.
